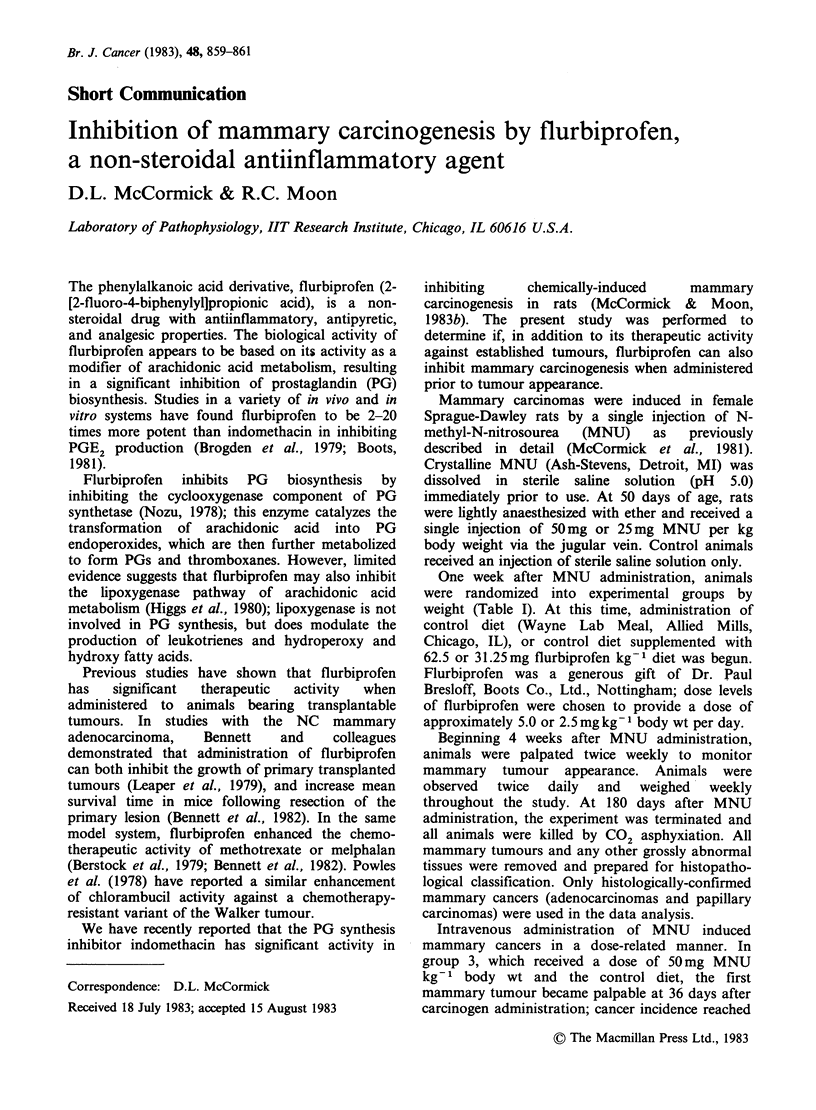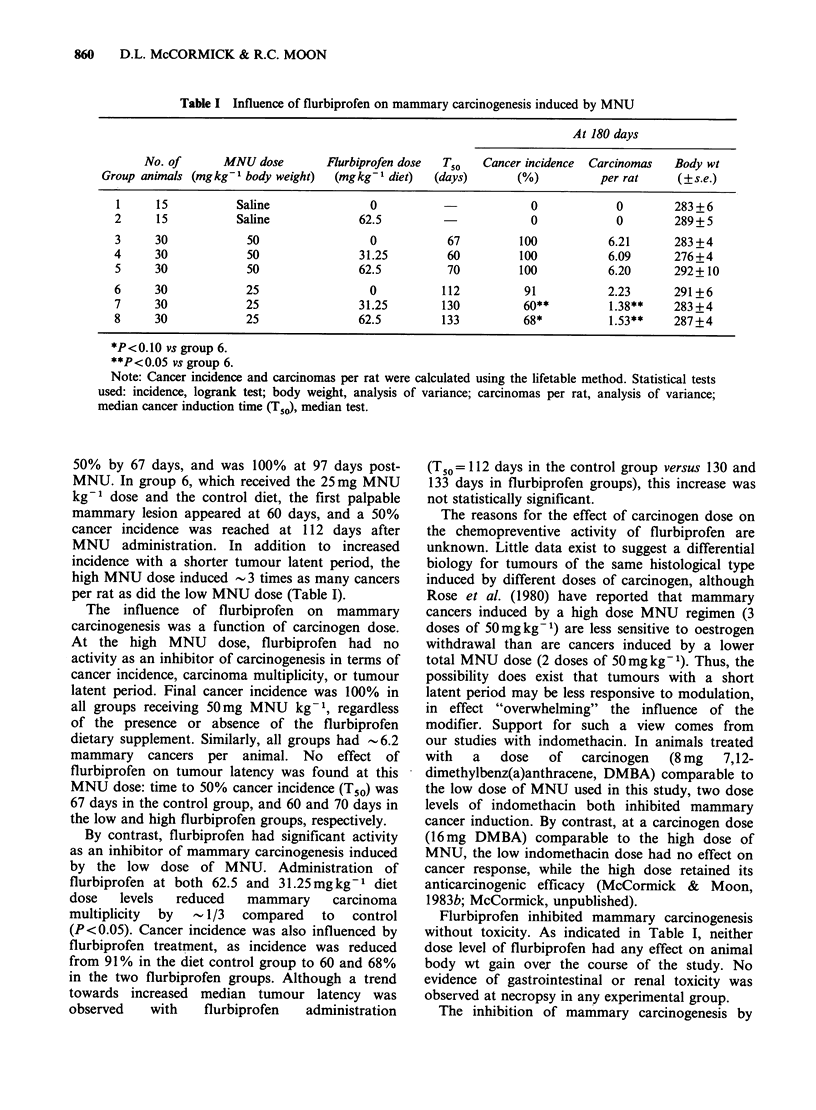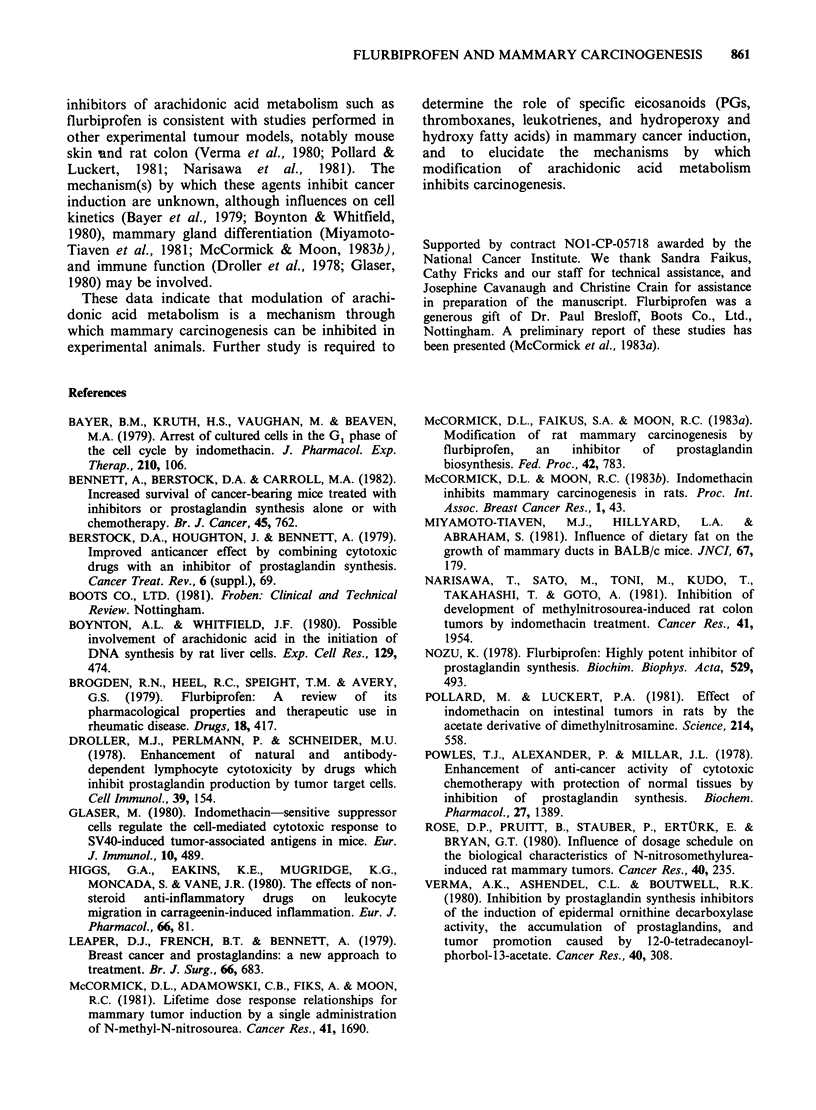# Inhibition of mammary carcinogenesis by flurbiprofen, a non-steroidal antiinflammatory agent.

**DOI:** 10.1038/bjc.1983.278

**Published:** 1983-12

**Authors:** D. L. McCormick, R. C. Moon


*Br. J. Cancer* (1983), **48**, 859–861

**Short Communication**

### Inhibition of mammary carcinogenesis by flurbiprofen, a non-steroidal antiinflammatory agent

D.L. McCormick & R.C. Moon

*Laboratory of Pathophysiology, IIT Research Institute, Chicago, IL 60616 U.S.A.*

The phenylalkanoic acid derivative, flurbiprofen (2-[2-fluoro-4-biphenylyl]propionic acid), is a non-steroidal drug with antiinflammatory, antipyretic, and analgesic properties. The biological activity of flurbiprofen appears to be based on its activity as a modifier of arachidonic acid metabolism, resulting in a significant inhibition of prostaglandin (PG) biosynthesis. Studies in a variety of *in vivo* and *in vitro* systems have found flurbiprofen to be 2–20 times more potent than indomethacin in inhibiting $PGE_2$ production (Brogden *et al.*, 1979; Boots, 1981).

Flurbiprofen inhibits PG biosynthesis by inhibiting the cyclooxygenase component of PG synthetase (Nozu, 1978); this enzyme catalyzes the transformation of arachidonic acid into PG endoperoxides, which are then further metabolized to form PGs and thromboxanes. However, limited evidence suggests that flurbiprofen may also inhibit the lipoxygenase pathway of arachidonic acid metabolism (Higgs *et al.*, 1980); lipoxygenase is not involved in PG synthesis, but does modulate the production of leukotrienes and hydroperoxy and hydroxy fatty acids.

Previous studies have shown that flurbiprofen has significant therapeutic activity when administered to animals bearing transplantable tumours. In studies with the NC mammary adenocarcinoma, Bennett and colleagues demonstrated that administration of flurbiprofen can both inhibit the growth of primary transplanted tumours (Leaper *et al.*, 1979), and increase mean survival time in mice following resection of the primary lesion (Bennett *et al.*, 1982). In the same model system, flurbiprofen enhanced the chemotherapeutic activity of methotrexate or melphalan (Berstock *et al.*, 1979; Bennett *et al.*, 1982). Powles *et al.* (1978) have reported a similar enhancement of chlorambucil activity against a chemotherapy-resistant variant of the Walker tumour.

We have recently reported that the PG synthesis inhibitor indomethacin has significant activity in inhibiting chemically-induced mammary carcinogenesis in rats (McCormick & Moon, 1983*b*). The present study was performed to determine if, in addition to its therapeutic activity against established tumours, flurbiprofen can also inhibit mammary carcinogenesis when administered prior to tumour appearance.

Mammary carcinomas were induced in female Sprague-Dawley rats by a single injection of N-methyl-N-nitrosourea (MNU) as previously described in detail (McCormick *et al.*, 1981). Crystalline MNU (Ash-Stevens, Detroit, MI) was dissolved in sterile saline solution (pH 5.0) immediately prior to use. At 50 days of age, rats were lightly anaesthesized with ether and received a single injection of 50 mg or 25 mg MNU per kg body weight via the jugular vein. Control animals received an injection of sterile saline solution only.

One week after MNU administration, animals were randomized into experimental groups by weight (Table I). At this time, administration of control diet (Wayne Lab Meal, Allied Mills, Chicago, IL), or control diet supplemented with 62.5 or 31.25 mg flurbiprofen $kg^{-1}$ diet was begun. Flurbiprofen was a generous gift of Dr. Paul Bresloff, Boots Co., Ltd., Nottingham; dose levels of flurbiprofen were chosen to provide a dose of approximately 5.0 or 2.5 mg $kg^{-1}$ body wt per day.

Beginning 4 weeks after MNU administration, animals were palpated twice weekly to monitor mammary tumour appearance. Animals were observed twice daily and weighed weekly throughout the study. At 180 days after MNU administration, the experiment was terminated and all animals were killed by $CO_2$ asphyxiation. All mammary tumours and any other grossly abnormal tissues were removed and prepared for histopathological classification. Only histologically-confirmed mammary cancers (adenocarcinomas and papillary carcinomas) were used in the data analysis.

Intravenous administration of MNU induced mammary cancers in a dose-related manner. In group 3, which received a dose of 50 mg MNU $kg^{-1}$ body wt and the control diet, the first mammary tumour became palpable at 36 days after carcinogen administration; cancer incidence reached

Correspondence: D.L. McCormick

Received 18 July 1983; accepted 15 August 1983

© The Macmillan Press Ltd., 1983

**Table I** Influence of flurbiprofen on mammary carcinogenesis induced by MNU

| Group | No. of animals | MNU dose (mg $kg^{-1}$ body weight) | Flurbiprofen dose (mg $kg^{-1}$ diet) | $T_{50}$ (days) | At 180 days: Cancer incidence (%) | At 180 days: Carcinomas per rat | At 180 days: Body wt (±s.e.) |
|---|---|---|---|---|---|---|---|
| 1 | 15 | Saline | 0 | — | 0 | 0 | 283±6 |
| 2 | 15 | Saline | 62.5 | — | 0 | 0 | 289±5 |
| 3 | 30 | 50 | 0 | 67 | 100 | 6.21 | 283±4 |
| 4 | 30 | 50 | 31.25 | 60 | 100 | 6.09 | 276±4 |
| 5 | 30 | 50 | 62.5 | 70 | 100 | 6.20 | 292±10 |
| 6 | 30 | 25 | 0 | 112 | 91 | 2.23 | 291±6 |
| 7 | 30 | 25 | 31.25 | 130 | 60** | 1.38** | 283±4 |
| 8 | 30 | 25 | 62.5 | 133 | 68* | 1.53** | 287±4 |

*$P<0.10$ *vs* group 6.
**$P<0.05$ *vs* group 6.
Note: Cancer incidence and carcinomas per rat were calculated using the lifetable method. Statistical tests used: incidence, logrank test; body weight, analysis of variance; carcinomas per rat, analysis of variance; median cancer induction time ($T_{50}$), median test.

50% by 67 days, and was 100% at 97 days post-MNU. In group 6, which received the 25 mg MNU $kg^{-1}$ dose and the control diet, the first palpable mammary lesion appeared at 60 days, and a 50% cancer incidence was reached at 112 days after MNU administration. In addition to increased incidence with a shorter tumour latent period, the high MNU dose induced ~3 times as many cancers per rat as did the low MNU dose (Table I).

The influence of flurbiprofen on mammary carcinogenesis was a function of carcinogen dose. At the high MNU dose, flurbiprofen had no activity as an inhibitor of carcinogenesis in terms of cancer incidence, carcinoma multiplicity, or tumour latent period. Final cancer incidence was 100% in all groups receiving 50 mg MNU $kg^{-1}$, regardless of the presence or absence of the flurbiprofen dietary supplement. Similarly, all groups had ~6.2 mammary cancers per animal. No effect of flurbiprofen on tumour latency was found at this MNU dose: time to 50% cancer incidence ($T_{50}$) was 67 days in the control group, and 60 and 70 days in the low and high flurbiprofen groups, respectively.

By contrast, flurbiprofen had significant activity as an inhibitor of mammary carcinogenesis induced by the low dose of MNU. Administration of flurbiprofen at both 62.5 and 31.25 mg $kg^{-1}$ diet dose levels reduced mammary carcinoma multiplicity by ~1/3 compared to control ($P<0.05$). Cancer incidence was also influenced by flurbiprofen treatment, as incidence was reduced from 91% in the diet control group to 60 and 68% in the two flurbiprofen groups. Although a trend towards increased median tumour latency was observed with flurbiprofen administration ($T_{50}=112$ days in the control group *versus* 130 and 133 days in flurbiprofen groups), this increase was not statistically significant.

The reasons for the effect of carcinogen dose on the chemopreventive activity of flurbiprofen are unknown. Little data exist to suggest a differential biology for tumours of the same histological type induced by different doses of carcinogen, although Rose *et al.* (1980) have reported that mammary cancers induced by a high dose MNU regimen (3 doses of 50 mg $kg^{-1}$) are less sensitive to oestrogen withdrawal than are cancers induced by a lower total MNU dose (2 doses of 50 mg $kg^{-1}$). Thus, the possibility does exist that tumours with a short latent period may be less responsive to modulation, in effect "overwhelming" the influence of the modifier. Support for such a view comes from our studies with indomethacin. In animals treated with a dose of carcinogen (8 mg 7,12-dimethylbenz(a)anthracene, DMBA) comparable to the low dose of MNU used in this study, two dose levels of indomethacin both inhibited mammary cancer induction. By contrast, at a carcinogen dose (16 mg DMBA) comparable to the high dose of MNU, the low indomethacin dose had no effect on cancer response, while the high dose retained its anticarcinogenic efficacy (McCormick & Moon, 1983*b*; McCormick, unpublished).

Flurbiprofen inhibited mammary carcinogenesis without toxicity. As indicated in Table I, neither dose level of flurbiprofen had any effect on animal body wt gain over the course of the study. No evidence of gastrointestinal or renal toxicity was observed at necropsy in any experimental group.

The inhibition of mammary carcinogenesis by inhibitors of arachidonic acid metabolism such as flurbiprofen is consistent with studies performed in other experimental tumour models, notably mouse skin and rat colon (Verma *et al.*, 1980; Pollard & Luckert, 1981; Narisawa *et al.*, 1981). The mechanism(s) by which these agents inhibit cancer induction are unknown, although influences on cell kinetics (Bayer *et al.*, 1979; Boynton & Whitfield, 1980), mammary gland differentiation (Miyamoto-Tiaven *et al.*, 1981; McCormick & Moon, 1983*b*), and immune function (Droller *et al.*, 1978; Glaser, 1980) may be involved.

These data indicate that modulation of arachidonic acid metabolism is a mechanism through which mammary carcinogenesis can be inhibited in experimental animals. Further study is required to determine the role of specific eicosanoids (PGs, thromboxanes, leukotrienes, and hydroperoxy and hydroxy fatty acids) in mammary cancer induction, and to elucidate the mechanisms by which modification of arachidonic acid metabolism inhibits carcinogenesis.

Supported by contract NO1-CP-05718 awarded by the National Cancer Institute. We thank Sandra Faikus, Cathy Fricks and our staff for technical assistance, and Josephine Cavanaugh and Christine Crain for assistance in preparation of the manuscript. Flurbiprofen was a generous gift of Dr. Paul Bresloff, Boots Co., Ltd., Nottingham. A preliminary report of these studies has been presented (McCormick *et al.*, 1983*a*).

**References**

BAYER, B.M., KRUTH, H.S., VAUGHAN, M. & BEAVEN, M.A. (1979). Arrest of cultured cells in the $G_1$ phase of the cell cycle by indomethacin. *J. Pharmacol. Exp. Therap.*, **210**, 106.

BENNETT, A., BERSTOCK, D.A. & CARROLL, M.A. (1982). Increased survival of cancer-bearing mice treated with inhibitors or prostaglandin synthesis alone or with chemotherapy. *Br. J. Cancer*, **45**, 762.

BERSTOCK, D.A., HOUGHTON, J. & BENNETT, A. (1979). Improved anticancer effect by combining cytotoxic drugs with an inhibitor of prostaglandin synthesis. *Cancer Treat. Rev.*, **6** (suppl.), 69.

BOOTS CO., LTD. (1981). *Froben: Clinical and Technical Review*. Nottingham.

BOYNTON, A.L. & WHITFIELD, J.F. (1980). Possible involvement of arachidonic acid in the initiation of DNA synthesis by rat liver cells. *Exp. Cell Res.*, **129**, 474.

BROGDEN, R.N., HEEL, R.C., SPEIGHT, T.M. & AVERY, G.S. (1979). Flurbiprofen: A review of its pharmacological properties and therapeutic use in rheumatic disease. *Drugs*, **18**, 417.

DROLLER, M.J., PERLMANN, P. & SCHNEIDER, M.U. (1978). Enhancement of natural and antibody-dependent lymphocyte cytotoxicity by drugs which inhibit prostaglandin production by tumor target cells. *Cell Immunol.*, **39**, 154.

GLASER, M. (1980). Indomethacin—sensitive suppressor cells regulate the cell-mediated cytotoxic response to SV40-induced tumor-associated antigens in mice. *Eur. J. Immunol.*, **10**, 489.

HIGGS, G.A., EAKINS, K.E., MUGRIDGE, K.G., MONCADA, S. & VANE, J.R. (1980). The effects of non-steroid anti-inflammatory drugs on leukocyte migration in carrageenin-induced inflammation. *Eur. J. Pharmacol.*, **66**, 81.

LEAPER, D.J., FRENCH, B.T. & BENNETT, A. (1979). Breast cancer and prostaglandins: a new approach to treatment. *Br. J. Surg.*, **66**, 683.

McCORMICK, D.L., ADAMOWSKI, C.B., FIKS, A. & MOON, R.C. (1981). Lifetime dose response relationships for mammary tumor induction by a single administration of N-methyl-N-nitrosourea. *Cancer Res.*, **41**, 1690.

McCORMICK, D.L., FAIKUS, S.A. & MOON, R.C. (1983*a*). Modification of rat mammary carcinogenesis by flurbiprofen, an inhibitor of prostaglandin biosynthesis. *Fed. Proc.*, **42**, 783.

McCORMICK, D.L. & MOON, R.C. (1983*b*). Indomethacin inhibits mammary carcinogenesis in rats. *Proc. Int. Assoc. Breast Cancer Res.*, **1**, 43.

MIYAMOTO-TIAVEN, M.J., HILLYARD, L.A. & ABRAHAM, S. (1981). Influence of dietary fat on the growth of mammary ducts in BALB/c mice. *JNCI*, **67**, 179.

NARISAWA, T., SATO, M., TONI, M., KUDO, T., TAKAHASHI, T. & GOTO, A. (1981). Inhibition of development of methylnitrosourea-induced rat colon tumors by indomethacin treatment. *Cancer Res.*, **41**, 1954.

NOZU, K. (1978). Flurbiprofen: Highly potent inhibitor of prostaglandin synthesis. *Biochim. Biophys. Acta*, **529**, 493.

POLLARD, M. & LUCKERT, P.A. (1981). Effect of indomethacin on intestinal tumors in rats by the acetate derivative of dimethylnitrosamine. *Science*, **214**, 558.

POWLES, T.J., ALEXANDER, P. & MILLAR, J.L. (1978). Enhancement of anti-cancer activity of cytotoxic chemotherapy with protection of normal tissues by inhibition of prostaglandin synthesis. *Biochem. Pharmacol.*, **27**, 1389.

ROSE, D.P., PRUITT, B., STAUBER, P., ERTÜRK, E. & BRYAN, G.T. (1980). Influence of dosage schedule on the biological characteristics of N-nitrosomethylurea-induced rat mammary tumors. *Cancer Res.*, **40**, 235.

VERMA, A.K., ASHENDEL, C.L. & BOUTWELL, R.K. (1980). Inhibition by prostaglandin synthesis inhibitors of the induction of epidermal ornithine decarboxylase activity, the accumulation of prostaglandins, and tumor promotion caused by 12-0-tetradecanoyl-phorbol-13-acetate. *Cancer Res.*, **40**, 308.